# Transvenous lead extraction: Experience of the Tandem approach

**DOI:** 10.1093/europace/euad331

**Published:** 2023-11-04

**Authors:** Zaki Akhtar, Christos Kontogiannis, Ahmed I Elbatran, Lisa W M Leung, Christoph T Starck, Zia Zuberi, Manav Sohal, Mark M Gallagher

**Affiliations:** Department of Cardiology, St George’s University Hospital, Blackshaw Road, Tooting, London SW17 0QT, UK; Department of Cardiology, St George’s University Hospital, Blackshaw Road, Tooting, London SW17 0QT, UK; Department of Cardiology, St George’s University Hospital, Blackshaw Road, Tooting, London SW17 0QT, UK; Department of Cardiology, Ain Shams University, Cairo, Egypt; Department of Cardiology, St George’s University Hospital, Blackshaw Road, Tooting, London SW17 0QT, UK; Department of Cardiothoracic Surgery, German Heart Centre, Berlin, Germany; Department of Cardiology, St George’s University Hospital, Blackshaw Road, Tooting, London SW17 0QT, UK; Department of Cardiology, St George’s University Hospital, Blackshaw Road, Tooting, London SW17 0QT, UK; Department of Cardiology, St George’s University Hospital, Blackshaw Road, Tooting, London SW17 0QT, UK

**Keywords:** Transvenous lead extraction, Lead extraction, Pacemaker extraction, Femoral extraction, Tandem approach, Non-laser transvenous lead extraction

## Abstract

**Aims:**

Transvenous lead extraction (TLE) is important in the management of cardiac implantable electronic devices but carries risk. It is most commonly completed from the superior access, often with ‘bail-out’ support via the femoral approach. Superior and inferior access may be used in tandem, which has been proposed as an advance in safety and efficacy. The aim of this study is to evaluate the safety and efficacy of the Tandem approach.

**Method:**

The ‘Tandem’ procedure entailed grasping of the targeted lead in the right atrium to provide countertraction as a rotational dissecting sheath was advanced over the lead from the subclavian access. Consecutive ‘Tandem’ procedures performed by a single operator between December 2020 and March 2023 in a single large-volume TLE centre were included and compared with the conventional superior approach (control) using 1:1 propensity score matching; patients were statistically matched for demographics.

**Results:**

The Tandem in comparison with the conventional approach extracted leads of much greater dwell time (148.9 ± 79 vs. 108.6 ± 77 months, *P* < 0.01) in a shorter procedure duration (96 ± 36 vs. 127 ± 67 min, *P* < 0.01) but requiring more fluoroscopy (16.4 ± 10.9 vs. 10.8 ± 14.9 min, *P* < 0.01). The Tandem and control groups had similar clinical (100% vs. 94.7%, *P* = 0.07) and complete (94.8% vs. 92.8%, *P* = 0.42) success, with comparable minor (4% vs. 6.7%, *P* = 0.72) and major (0% vs. 4%, *P* = 0.25) complications; procedural (0% vs. 1.3%, *P* = 1) and 30-day (1.3% vs. 4%, *P* = 0.62) mortality were also similar.

**Conclusion:**

The Tandem procedure is as safe and effective as the conventional TLE. It can be applied to leads of a long dwell time with a potentially shorter procedure duration.

## Graphical Abstract

**Graphical Abstract:**
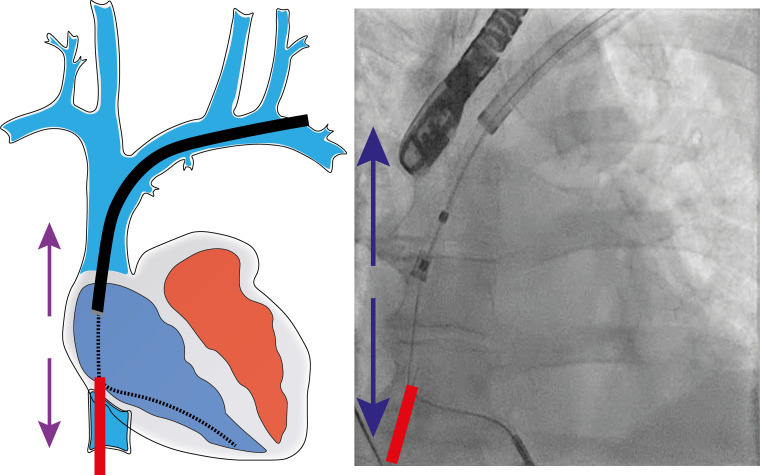

**What’s new?**

- This is the largest Tandem technique series in Europe.
- We compared the Tandem technique with the conventional superior approach using 1:1 propensity score matching.
- The Tandem technique is safe and effective in leads of a long dwell time.
- The procedure duration is shorter with the Tandem technique, albeit with an extended fluoroscopy exposure.

## Introduction

Transvenous lead extraction (TLE) has become integral in the management of cardiac implantable electronic devices (CIEDs). A rising number of CIED implants and an expanding indication have fuelled increasing demand for TLE. Expert consensus has recommended hardware extraction for infection and non-infection indications.^1^ Fibrosis and calcification encapsulating chronically implanted leads can make TLE a challenging process. This has led to the development of specialized techniques and equipment, including rotational and laser-powered sheaths, to free the lead from the binding tissue. Although effective, these methods carry a risk of major morbidity and death; injury to the superior vena cava (SVC) is a potentially catastrophic complication.^2^ The vulnerability of the SVC may partly be from the acute angulation at the transition from the innominate vein; during TLE, the dissecting sheath tip often fails to remain coaxial at this angulation and may advance into the SVC wall.

Despite the advances in techniques, complete lead removal cannot always be achieved from superior access. Specialized snare tools introduced via the femoral vein can be used to complete the extraction; this ‘bail-out’ approach is required in 5% of cases^3^ which are often challenging cases with longer lead dwell time and a high number of leads to extract.^4^ A small number of operators have utilized the femoral access as the primary route for TLE with clinical success rates of 98%.^5^ These two TLE routes have also been used in ‘Tandem’.^6,7^ This advanced technique provides geometric advantages and a theoretical low risk of SVC injury; however, its application is currently limited to very few institutes.^6,7^ In this study, we report the outcomes of non-laser TLE used in conjunction with the femoral snare, the initial experience of the ‘Tandem’ approach, from a single high-volume European centre.

## Method

One high-volume operator adopted the Tandem technique for all except the lowest-risk targeted leads (lead dwell time <24 months) in a consecutive series between December 2020 and March 2023. Patient and procedural data were collected prospectively. Historical data of TLE procedures utilizing a superior rotational approach (with femoral bail-out where necessary) performed by the same operator were also collected and used as the ‘control’ group. The operator was already experienced with >300 non-laser TLE procedures when the ‘control’ group was treated. Primary outcomes included major complication, procedural mortality, 30-day mortality, complete success (per lead), and clinical success (per patient); secondary outcomes comprised minor complication, procedure duration, fluoroscopy time, and the occurrence of the dissecting sheath reaching the distal portion of the lead. The study was in accordance with the local institutional review board guidelines and complies with the principles of the Declaration of Helsinki.

All extraction procedures were defined and performed in accordance with the Heart Rhythm Society (HRS)^8^ and European Heart Rhythm Association (EHRA) consensus.^1^ For all TLE procedures, a cardiac surgeon remained on stand-by with a perfusionist while the procedure was performed in the cardiac catheterization suite; femoral venous access with invasive arterial pressure monitoring was prepared prior to extraction, and a temporary pacing system was positioned when required. The ‘traditional/conventional’ TLE procedure followed a standardized pattern: after excising the generator and leads free from the pocket, the fixation mechanism of the lead was withdrawn when possible. The leads were then cut and a locking stylet (Liberator, Cook Medical, USA) was deployed, followed by a compression coil (OneTie, Cook Medical, USA). A rotational dissecting tool (Evolution, Evolution RL, Cook Medical, USA) was then directed over the lead to dissect it free from the adhesions, assisted by traction applied to the locking stylet.

The ‘Tandem’ procedure also followed a standard protocol. As the first operator dissected the implant site, the second operator secured femoral venous access and used it to advance the Needle’s Eye Snare (NES) introducer sheath (Cook Medical, USA) to the right atrium (RA). Through this introducer, an inner curved sheath (Cook Medical, USA) harbouring the snare was positioned in the RA; the curved tip of the inner sheath improved the reach of the snare comparatively with the standard non-curved sheath. After freeing the hardware from the pocket, the leads were mobilized and the fixation mechanism was retracted when possible. After deployment of the locking stylet, the NES was used to grasp the targeted lead in the RA. Both operators then exerted firm traction, in opposing directions, on the lead to achieve balance, so that the point of interaction between snare and lead remained in the lower part of the RA. Traction and countertraction were maintained, while a rotational dissecting sheath (Evolution, Evolution RL, Cook Medical, USA) was advanced over the lead, cutting it free from the encapsulating adhesions, until it reached the NES. The lead was then released from the snare, and the rotational tool continued to dissect towards the lead tip using traction and countertraction. If this failed to free the lead tip from the encapsulation, the rotational mechanism was activated as traction is applied to engulf the lead further into the sheath; as the lead is engulfed, the rotating mechanism peels the adhesions away to free the lead tip and complete the extraction (*Video*). This technique prohibits any forward force from the sheath being applied to the heart, minimizing the risk of myocardial perforation.

### Definitions

In accordance with the EHRA and HRS consensus, complete procedural success was defined as the removal of all lead components from the vasculature without causing a fatal or disabling complication.^1,8^ A complication was the undesired consequence of the extraction procedure causing suffering, disability, prolonged hospital stay, or requirement for further therapy, and it was subcategorized into major or minor. A complication was considered ‘major’ if it caused disability or death or if required major surgical intervention to prevent disability or death; a minor complication was classified as an undesired consequence of the procedure which does not limit the patient’s function or cause death. Any death occurring on the procedural day, or a later death that arose from a procedure-related complication, was recorded as a procedure-related fatality.

### Statistics

Categorical variables were expressed as a number and percentage. Continuous variables were reported as mean ± standard deviation or median with interquartile range (IQR). To allow a comparison between the ‘Tandem’ and traditional TLE approach, propensity score matching was performed. A propensity score was calculated for all eligible patients undergoing lead extraction. Logistic regression with the use of Tandem procedure as the binary outcome and baseline variables were used as covariates for estimating the propensity score. Propensity matching was performed in a 1:1 fashion using the nearest neighbour approach with a two decimal calliper.

Procedures were matched for patient age, gender, body mass index (BMI), left ventricle ejection fraction (LVEF), comorbidities (diabetes, hypertension, ischaemic heart disease, and chronic kidney disease), infection as the indication for extraction, pacemaker vs. implantable cardioverter defibrillator (ICD), and operator and operating theatre vs. cardiac catheterization suite. Seventy-five procedures in the Tandem group were matched to 75 procedures from the historical database.

Dichotomous categorical data were analysed using McNemar’s test, while continuous variables were analysed using the paired Student’s *t*-test. Statistical analysis was performed using SPSS statistical software, version 28 (IBM Corp., Chicago, IL, USA).

## Results

Over the study period, there were 75 ‘Tandem’ TLE procedures performed in mostly male patients (72%), aged 67.9 ± 16.1 years with a BMI of 26.6 ± 4.8 kg/m^2^. In this cohort, 45 patients had hypertension, 13 diabetes, and 23 ischaemic heart disease with an average LVEF of 45.5 ± 11.8%. In these 75 patients, there were 170 leads in total, of which 153 were targeted for extraction with a non-infectious indication (60%) and a dwell time of 148.9 ± 79 months; the majority was active fixation leads (62.1%) positioned in the right ventricle (RV) (55.6%) in a dual-chamber system (43%). Of the targeted 153 leads, 57 (37.3%), 78 (50.9%), and 18 (11.8%) leads had a dwell time of <10, 10–20, and >20 years, respectively; in total, only 13 leads were extracted with manual traction all of which were <10 years in age.

In the Tandem group, the 13-french Evolution RL sheath with the 13 mm NES were used for the majority of leads extracted (46% and 93%, respectively); 94% of targeted leads were successfully snared (100% RA, 92% RV, 90% LV). Additional tools were required to perform the jugular pull-through in only six leads for completion; the Tandem forms a normal part of the jugular pull-through technique.^9^ Complete procedural success was achieved in 95% of leads and 100% clinical success with 4% minor complication in procedures lasting 96 ± 36 min requiring 16.4 ± 10.9 min of fluoroscopy; there were no major complications or procedural mortality.

The Tandem and non-Tandem groups were statistically matched for demographics with propensity score matching. There was a statistically similar proportion of male patients of a similar age, with a comparable BMI, LVEF, comorbidities, and infection indication for extraction (*Table 1*). The Tandem procedure in comparison with the control was used to extract leads of a much longer dwell time (148.9 ± 79 vs. 108.6 ± 77 months, *P* < 0.01) in a shorter procedure duration (96 ± 36 vs. 127 ± 67 min, *P* < 0.001) but requiring an extended fluoroscopy time (16.4 ± 10.9 vs. 10.8 ± 14.9 min, *P* < 0.001).

**Table 1:** A comparison of the patient series for whom the Tandem method was used against the conventional lead extraction group matched by propensity score

Variable	Tandem	Control	*P*-value
Patients (n=)	75	75	
Demographics			
Sex (male), *n* (%)	54 (72)	50 (67)	0.56
Age (years), mean ± SD	67.9 ± 16.1	68.3 ± 16.3	0.85
BMI (kg/m^2^), mean ± SD	26.6 ± 4.8	26.7 ± 5.3	0.92
LVEF (%), mean ± SD	45.5 ± 11.8	45.1 ± 11.6	0.83
Hypertension, *n* (%)	45 (60)	41 (55)	0.57
Diabetes mellitus, *n* (%)	13 (17)	12 (16)	0.99
Chronic kidney disease, *n* (%)	9 (12)	6 (8)	0.61
Ischaemic heart disease, *n* (%)	23 (31)	27 (36)	0.61
Non-ischaemic cardiomyopathy, *n* (%)	24 (32)	18 (24)	0.34
Cardiac surgery, *n* (%)	12 (16)	14 (19)	0.83
Infection as indication for TLE, *n* (%)	30 (40)	32 (43)	0.86
Defibrillator system, *n* (%)	35 (46.7)	34 (45.3)	0.99
Targeted leads			
n=	153	139	
Lead dwell time (months), mean ± SD	148.9 ± 79	108.6 ± 77	**<0**.**01**
Active lead fixation mechanism, *n* (%)	95 (62)	87 (63)	0.93
Defibrillator leads (%)	37 (24.1)	35 (25.2)	0.84
Dual-coil leads (%)	11 (7.2)	10 (7.2)	0.99
Liberator locking stylet, *n* (%)	141 (92)	119 (86)	0.07
Bulldog lead extender, *n* (%)	7 (5)	11 (8)	0.24
Rotational dissecting sheath use, *n* (%)	140 (92)	132 (95)	0.24
Additional tools (per lead)	6 (3.9)	16 (11.5)	**0**.**01**
Procedural outcomes			
General anaesthesia, *n* (%)	73 (97)	68 (91)	0.18
Rotational tool reaching distal lead tip (per lead), *n* (%)	147 (96%)	54 (37%)	**<0**.**01**
Procedure duration (minutes), mean ± SD	96 ± 36	127 ± 67	**<0**.**01**
Fluoroscopy time (minutes), mean ± SD	16.4 ± 10.9	10.8 ± 14.9	**<0**.**01**
Radiation dose area product (Gy.cm^2^)	4.8 ± 3.9	1.1 ± 1.7	**<0**.**01**
Complete success (per lead), *n* %	145 (94.8)	129 (92.8)	0.42
Clinical success, *n* %	75 (100)	71 (94.7)	0.13
Major complication, *n* (%)	0	3 (4)	0.25
Minor complication, *n* (%)	3 (4)	5 (6.7)	0.72
Procedural mortality, *n* (%)	0	1(1.3)	1
Thirty-day mortality, *n* (%)	1(1.3)	3 (4)	0.62

BMI, body mass index; LVEF, left ventricular ejection fraction; TLE, transvenous lead extraction.

Clinical success was statistically similar between the Tandem and the control group (100% vs. 94.7%, *P* = 0.13), as were complete procedural success per lead (94.8% vs. 92.8%, *P* = 0.42), minor complications (4% vs. 6.7%, *P* = 0.72), and major complications (0% vs. 4%, *P* = 0.25) (*Table 1*). There was no difference between the Tandem and control in perioperative mortality (0% vs. 1.3%, respectively, *P* = 1) or 30-day mortality (1.3% vs. 4%, *P* = 0.62) (*Table 2*). There were a significantly higher proportion of cases in which the rotational dissecting sheath reached the distal lead end with the Tandem (96%) comparatively with the control (37%) (*P* < 0.01).

**Table 2:** Summary of mortality

Patient	Cohort	Device	Targeted leads	Procedural indication	Dwell time of oldest lead (months)	Complication	Detail
66-year-old male	Tandem	CRT-D	5	Infection	161	Minor	Bleeding from pocket site. Died 7 days post-procedure from sepsis
90-year-old female	Control	Pacemaker	2	Infection	166	Nil	No procedural complication. Died 23 days post-procedure from infection
85-year-old male	Control	Pacemaker	1	Infection	244	Nil	No procedural complications. Died 28 days post-procedure from sepsis
61-year-old female	Control	Pacemaker	3	Infection	208	Major; mortality	SVC tear at the SVC–RA junction, resulting in pericardial tamponade requiring sternotomy and repair of the injury. Patient died 2 days post-procedure

CRT-D, cardiac resynchronization therapy-defibrillator; RA, right atrium; SVC, superior vena cava.

## Discussion

In this study, we evaluated the non-laser ‘Tandem’ procedure in which we have demonstrated that this is a safe and effective technique. There were no major complications or mortality while achieving a high clinical and complete success rate. This technique provides an additional dimension to TLE, improving safety and achieving high efficacy.

**Figure 1:**
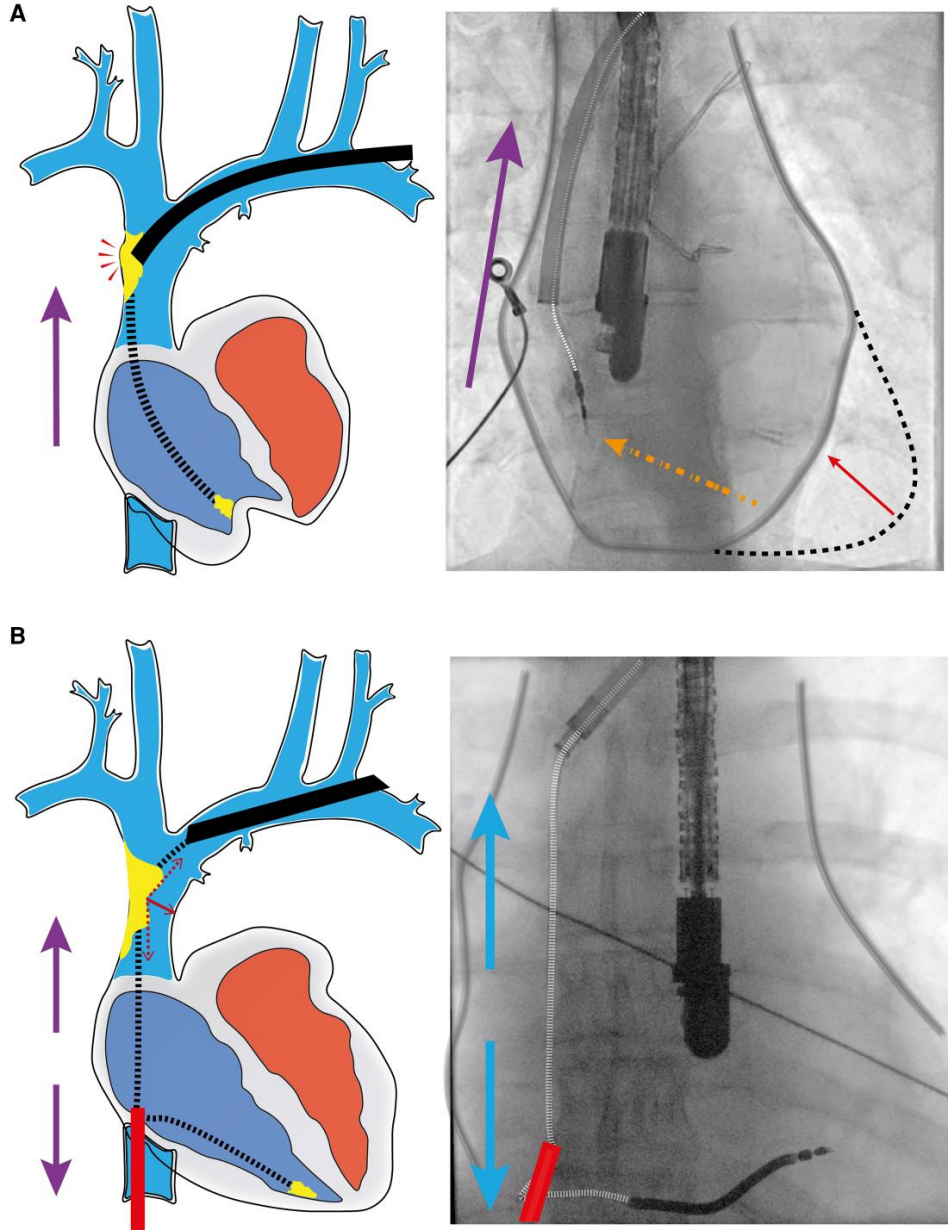
The Tandem procedure illustrated. (*A*) Without the countertraction provided by the femoral snare, the traction applied from the superior access is relayed to the right ventricle (RV). This results in invagination of the RV which increases the risk of avulsion injury. Furthermore, the dissecting sheath does not have the firm rail to steer clear of the superior vena cava wall and inadvertently can tear this vessel. (*B*) With the countertraction from the femoral snare (Tandem), the traction force is redirected to the snare, and the rail is firmly straightened. This pulls the lead adhered to the SVC, away from the wall, and steers the dissecting sheath towards the inferior vena cava.

**Figure 2:**
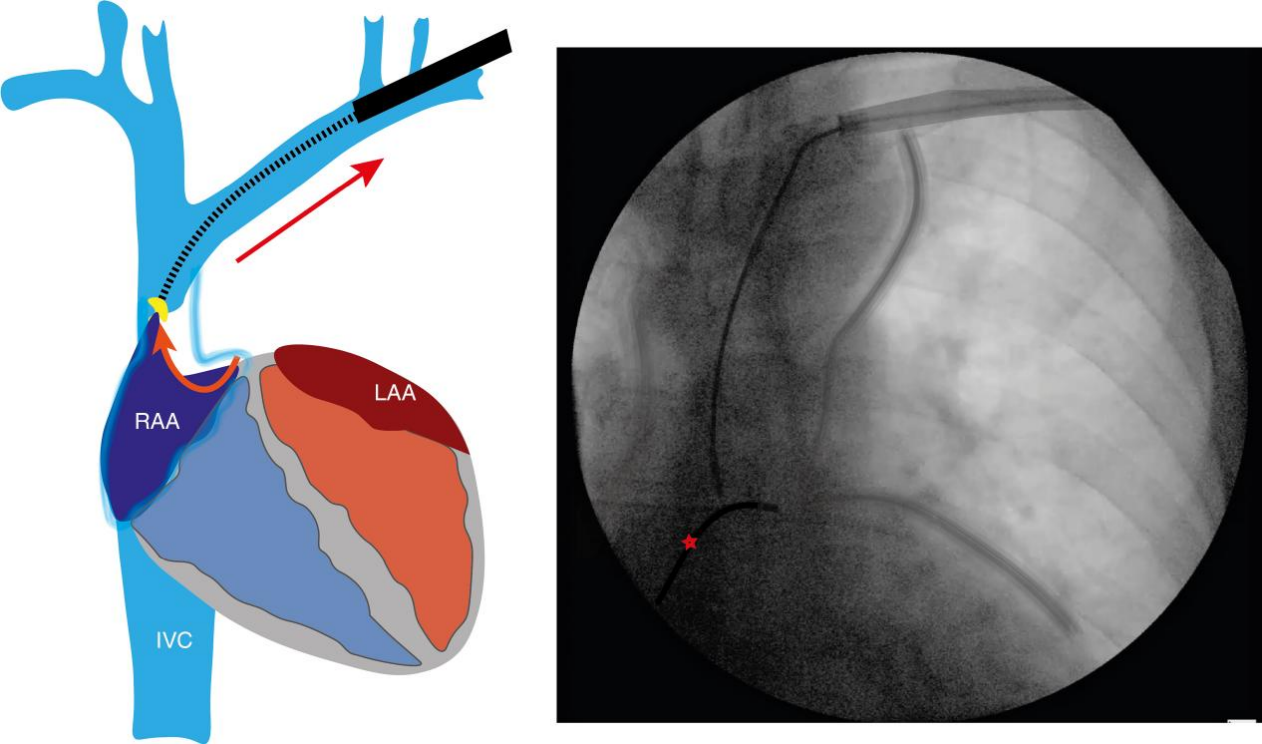
Invagination of the right atrium (RA). Without the Tandem approach, there is invagination of the right atrial appendage (curved arrow) as the atrial lead is pulled with excessive superior traction during an attempt to advance the rotational dissecting sheath (straight arrow). In this case, there was avulsion injury of the RA requiring an emergency sternotomy. On fluoroscopy, note the significant displacement of the temporary pacing wire (red star), resulting from the invagination of the myocardium as superior traction is applied. IVC, inferior vena cava; LAA, left atrium appendage; RAA, right atrium appendage.

A good rail for the advancement of the dissecting sheath forms the basis of a safe and effective TLE (*Figure 1*). Traditionally, this rail is generated by the unidirectional upward traction applied on the targeted lead. This has fundamental limitations. The geometry is unfavourable; unopposed traction applied on the targeted lead from superior access results in abnormal stress being transmitted directly to the SVC and the heart (*Figure 2*), increasing the risk of injury.^10^ Unidirectional traction on the lead is limited by the risk of damage to the lead or to the cardiovascular body and may therefore not be sufficient to provide a straight, taut rail for the dissecting sheath. Without a firm rail, the sheath may not remain intraluminal as it navigates the innominate–SVC junction, risking perforation and catastrophic haemorrhage. Transmission of traction to the heart can also be dangerous. The traction force applied on the encapsulating tissue is dynamic; the lead may be free to slide through the adhesions, or the binding sites themselves may move with the lead, allowing the force to transmit to the lead tip, potentially causing myocardial invagination or avulsion with haemodynamic compromise.

**Figure 3:**
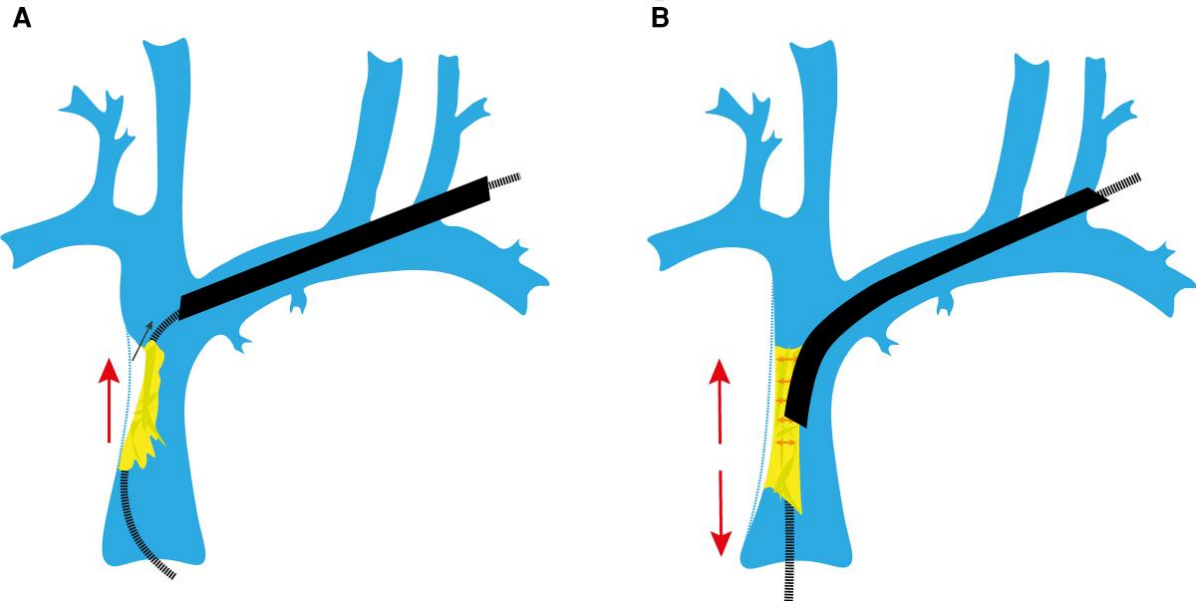
Diagram demonstrating the geometric relationship of the lead with the superior vena cava wall. (*A*) Superior only traction (large arrow) applies direct pull on the entire length of the lead and the encapsulation that surrounds it. This can cause the tissue to ‘bunch up’, increasing the difficulty of dissection. The force that is transmitted to the lead tip can injure the heart by avulsion. Because the force is relatively weak, it fails to adequately pull the lead and binding tissue away from the SVC wall, instead pulling the whole structure superiorly which can bring the SVC wall in to firm contact with the dissecting sheath. (*B*) With the Tandem technique, the firm countervailing superior–femoral force improves the geometry: the binding tissue is stretched to permit a clean dissection by the sheath while the lead pulled medially along with its binding tissue, away from the SVC wall; with the balancing of applied forces, there is minimal distortion of the SVC.

Snaring the lead via the femoral access with the NES has significant advantages. With the locking stylet deployed, it transforms the locking stylet into the rail for the dissecting sheath, capable of bearing a traction load in excess of 7 kg.^11^ The critical step is to grip the lead at a point where the locking stylet lies within the lumen and pull it in to the NES sheath. This kinks the locking stylet within the lead lumen and secures it in position; without this step, the lead can disintegrate without much effort.^11^ In this configuration, it is able to resist several kilograms of maintained countertraction, straightening the lead and holding it firm—key characteristics of a reliable rail.^11^ This forces the dissecting sheath to remain coaxial, preventing it from cutting into the SVC wall. The simultaneous traction and countertraction also improves the geometric relationship between the lead and SVC. The balanced opposing forces pull the lead away from the vessel wall and towards the lumen (*Figure 3*). This minimizes contact of the sheath with the wall during dissection and in turn reduces the risk of SVC injury.^7^ The snare also transfers the point of tension away from the heart during superior traction and rests it upon itself. This reduces the risk of avulsion. It probably is most important when the rotational sheath first enters the venous system superiorly; resistance to the entry of the dissecting sheath beneath the clavicle can require substantial countertraction to overcome the resistance and advance the dissecting sheath. In our experience, the likelihood of avulsion injury is high at this stage (*Figure 2*).

The benefits of the Tandem procedure were most evident among patients with leads of the longest dwell time. Lead dwell time is a significant variate associated with incomplete success, complications, and adverse outcomes.^12^ In the TLE risk stratification ELECTRa Registry Outcome Score (EROS), only patients with pacemaker leads of >15 years and ICD leads of >10 years dwell time were associated with the highest risk of complications and mortality and a lower success rate.^13^ This is logical as longer dwell time enhances the strength of lead encapsulation in the vasculature and the difficulty it may pose to extraction. Encapsulating tissue begins as a thrombus bound to the lead, which over time organizes and transforms into a collagenous capsule. With time this thickens and mineralizes leading to a calcified dense binding sheath.^14^ In our study, the Tandem group had a far greater lead dwell time than the traditional TLE cohort, yet a similar rate of complete technical success and clinical success was achieved without any major complication or mortality. We believe that this is due in part to the stretching and straightening of the encapsulating tissue produced by the geometry of forces in the Tandem method. This subtle factor is crucial to achieving a clean dissection of the adhesions; a straightened and stretched tissue is easier to cut. Without the Tandem, the encapsulating tissue can bunch up, making it more difficult to cut cleanly.

Clinical success and complete procedural success in our series was consistent with that of Muhlestein *et al.*^6^ (96.2% and 92.1%, respectively), who also used the same methodology of non-laser ‘Tandem’. This validates the efficacy of the technique as the results are reproducible and comparable with large conventional TLE series including PROMET^15^ and ELECTRa.^12^

There are important differences between our study and that of Muhlestein *et al*. We performed a comparison between the ‘Tandem’ and the conventional rotational sheath TLE method, in which major variables including primary operator and patient demographics were matched. Our cohort had a greater lead dwell time (12.3 years) than that of Muhlestein *et al.* (9.8 years). Also, a substantial majority (67%) of the leads extracted by the Tandem method in our series had a dwell time of >10 years compared with 43% in those reported by Muhlestein *et al*.^6^ Our study validates the outcomes of that group, extends these findings to an older patient cohort with longer lead dwell time, and provides some insight when compared with the standard TLE method.

Surprisingly, Muhlestein *et al.* reported three cases of pericardial tamponade and an overall major complication rate of 3.1%.^6^ We did not experience any significant complications, despite applying an identical series of steps. The mechanism of injury in those cases has not been detailed. It could be associated with failure of the rotational sheath to reach the lead tip to complete the extraction after the lead has been released from the NES. Forceful traction to free the lead from the endocardium can cause myocardial injury with a resulting pericardial effusion. This would be consistent with the PROMET series which had identified RV injuries to be the predominant major complication in association with the rotational dissecting sheath.

In our series, the dissecting sheath reached the lead tip on 96% of the leads extracted; with the traditional extraction, this occurred in 37% of leads targeted (*P* < 0.01). This is a significant endpoint often overlooked. Having the extraction sheath reach the lead tip signifies effective dissection of the adhesion tissue and permits dissection of the lead tip from the myocardium which is often the most securely bound,^14^ especially with passive fixation.^16^ This is more likely to be safe as lead extractions performed with failure of the sheath to advance to the distal end entail significant traction force to ‘rip’ the lead out of their endocardial encapsulations. Having the sheath reach past the SVC also allows maintenance of the vascular access which is crucial to overcome vascular occlusions when upgrading the hardware; up to 26% of extraction referrals do have venous occlusions,^17^ and venous occlusion is an indication for TLE.^18^

An alternative explanation of the tamponades seen in previous Tandem experiences would be the occurrence of atrial perforation by the ‘threader’ of the Needle’s Eye Snare. Having been alerted by previous experience, we ensured that the deployment of the ‘threader’ through the ‘Needle’s Eye’ was slow and cautious in all cases. We also favoured the smaller (13 mm) size of NES which we believe reduced the risk of perforation; the larger alternative (20 mm) has a longer ‘threader’ which is more likely to cause injury.

**Figure 4:**
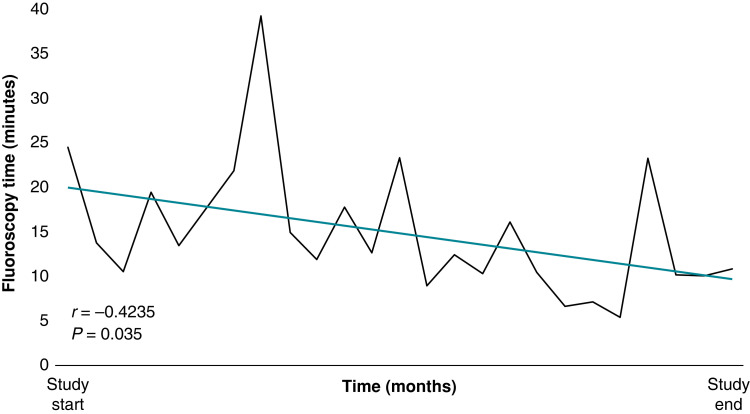
Line graph highlighting the fluoroscopy time over the course of the study period. Average fluoroscopy time (minutes) per month in chronological order over the study period. Fluoroscopy exposure reduces with increasing experience (*P* = 0.035); there is a learning curve associated with the Needle’s Eye Snare (NES) (five outlier cases were removed for the purpose of this analysis).

Muhlestein *et al.* were unable to provide fluoroscopy time for their cases. Our study demonstrated that the ‘Tandem’ extraction increased fluoroscopy time. This is expected as it is an additional segment of the conventional TLE procedure which is fluoroscopy dependent; it is required to visualize the skeleton of the NES grasp the lead, which can be challenging. It is especially a concern in the beginning of the learning curve when the use of the snare is a novelty; with developing familiarity of the tool, the fluoroscopy dependency is expected to shorten (*Figure 4*). Notably, the procedure time was overall reduced with the added use of the NES, a secondary effect of the Tandem for several reasons. The combined opposing traction forces imposed on the lead could have stretched the body, shrinking the lead diameter and improving the lead’s ability to escape the binding tissue which may reduce the overall dissection time.^19^ The firm rail provided by the Tandem may improve the efficiency of the procedure by reducing the need for complementary extraction steps. It also readies the ‘bail-out’ phase from the beginning, achieved with reduced effort comparatively with attempting the snaring towards the end which is challenging and time-consuming.

As with all techniques, there are inherent limitations with the ‘Tandem’ procedure. The obvious limitation is the challenge of successfully grasping the lead, particularly the difficulty of doing so without disturbance to bystander leads that are not targeted for extraction. The NES requires careful attention to the geometry of the interaction between lead and snare. This orientation is difficult to achieve in two-dimensional fluoroscopy imaging. Occasionally, the hooking of the lead with the NES can prove impossible as the lead can be tethered to the heart wall with no free lead portion to be ‘hooked’; this was the case in a very small proportion of leads in our study (8%) where complete procedural success was not achieved. Conversely, we were able to snare 100% of the targeted RA leads and complete their extraction with the Tandem technique; deployment of the atrial lead in the RA appendage results in a loop that compliments the NES for snaring. Femoral approach is also associated with a higher complication risk^12^; the NES requires the 16-french outer sheath, and there is risk of vascular injury, bleeding, and infection. There are also economic limitations: the use of two extraction tools increases the financial costs of a single procedure. Subsequently, the use of the ‘Tandem’ procedure could be reserved for the challenging cases which may include passive fixation ICD leads of a long dwell time (>10 years). Zabek *et al.*^20^ found that dual-coil ICD leads with a passive fixation and >10 years dwell time significantly increased the complexity of the extraction. Although the success rate was high, multiple tools and techniques were required, including the femoral approach and there was a notable trend towards a higher complication rate. Patient’s with systemic infection,^12^ an unfavourable anatomy,^21^ and cases of superior venous occlusion that require access preservation may also benefit from the Tandem technique, while pacemaker leads of a short dwell time in patients with a non-infectious TLE indication may be appropriately served with the conventional TLE approach.

## Limitations

This study compared the ‘Tandem’ with the contemporary method of TLE based on a single centre with a small number of operators, with their own specific techniques. Additionally, the main operator was already highly experienced prior to the Tandem, while the inexperienced second operator gained concentrated experience of the NES with the Tandem. This may contribute to the efficacy and safety of the Tandem, and consequently, our findings are not generalizable. The non-randomization nature of our study is an important limitation; a randomized study involving more operators would be required to reduce the risk of potential of technique or experience bias. The population size was not large enough to detect low incidences of complications. All procedures were performed with rotational dissecting sheaths (Evolution RL) for TLE, and our results may not be applicable to other extraction methods; however, successful application of the Tandem technique using the laser sheath has been reported.^7^

## Conclusion

The Tandem procedure is safe and effective as a primary TLE technique. It can be applied with a favourable profile to leads of long dwell time, with the potential to reduce procedure duration.

## Supplementary Material

euad331_Supplementary_Data

## Data Availability

Data are on file and available on reasonable request.
