# Supplementary material for: Transvenous lead extraction: Experience of the Tandem approach
Source: Europace. 2023 Nov 4;25(11):euad331. doi: 10.1093/europace/euad331 (PMC10903175; doi:10.1093/europace/euad331)
Supplement: euad331_Supplementary_Data [file euad331_Supplementary_Data.docx]

To allow a comparison between the ‘Tandem’ and traditional TLE approach (control), propensity score matching was performed. A propensity score was calculated for all eligible patients undergoing lead extraction. Logistic regression with use of tandem procedure as the binary outcome and baseline variables were used as covariates for estimating the propensity score. Propensity matching was performed in a 1:1 fashion using the nearest neighbour approach with a two decimal caliper.

The two groups were matched for age, gender, body mass index, comorbidities (diabetes, hypertension, ischaemic heart disease, chronic kidney disease), the type of device (pacemaker vs ICD), the operator (cardiologist vs cardiac surgeon), operating theatre vs cath lab, infection as an indication for lead extraction and lead dwell time. Statistical analysis was performed using SPSS statistical software, version 28 (IBM Corp., Chicago, IL, USA).

**Supplementary table 1:** Comparison of outcomes

| **Procedural outcomes (lead dwell time matched)** | Tandem | Control | p-value |
| --- | --- | --- | --- |
| Rotational tool reaching distal lead tip (per lead), (%) | 96% | 36% | **<0.01** |
| Procedure duration (minutes), mean± SD | 96 ± 36 | 126.2 ± 67 | **<0.01** |
| Fluoroscopy time (minutes), mean ± SD | 16.4 ± 10.9 | 10.6 ± 15 | **<0.01** |
| Complete success (per lead), % | 94.8% | 92% | 0.34 |
| Clinical success % | 100% | 95.9% | 0.25 |
| Major complication (%) | 0 | 4.1% | 0.25 |
| Minor complication (%) | 4% | 8.1% | 0.3 |
| Procedural mortality (%) | 0 | 4.1% | 0.25 |
| Thirty-day mortality (%) | 1.3% | 5.4% | 0.37 |
